# Severe Neuropsychiatric Reaction in a Deployed Military Member after Prophylactic Mefloquine

**DOI:** 10.1155/2011/350417

**Published:** 2011-09-08

**Authors:** Alan L. Peterson, Robert A. Seegmiller, Libby S. Schindler

**Affiliations:** ^1^Department of Psychiatry, University of Texas Health Science Center, San Antonio, TX 78229, USA; ^2^59th Medical Operations Squadron, Wilford Hall Medical Center, San Antonio, TX 78236, USA

## Abstract

Recent studies of military personnel who have deployed to Iraq and Afghanistan have reported a number of combat-related psychiatric disorders such as posttraumatic stress disorder, depression, and traumatic brain injury. This case report involves a 27-year-old male active-duty US military service member who developed severe depression, psychotic hallucinations, and neuropsychological sequelae following the prophylactic use of the antimalarial medication mefloquine hydrochloride. The patient had a recent history of depression and was taking antidepressant medications at the time of his deployment to the Middle East. Psychiatrists and other health care providers should be aware of the possible neuropsychiatric side effects of mefloquine in deployed military personnel and should consider the use of other medications for malaria prophylaxis in those individuals who may be at increased risk for side effects.

## 1. Introduction

The stress of military combat can result in a wide array of psychiatric conditions [1]. Posttraumatic stress disorder, depression, and traumatic brain injury are three of the most commonly reported psychiatric conditions reported in studies of multinational military personnel who have served in the wars in Iraq and Afghanistan [2–4]. Although most combat-related psychiatric conditions are thought to be related to events occurring on the military battlefield, neuropsychiatric reactions can also be related to a number of other deployment-related factors such as environmental exposures [5], chemical warfare agents [6], immunizations [7], and the use of prophylactic medications [8].

Mefloquine was initially developed as a treatment for malaria by the US Army in the 1960s [9]. It was introduced for the treatment of malaria in the late 1970s and became available as a prophylactic drug in 1985. Mefloquine is indicated for the prophylaxis and treatment of *Plasmodium vivax* and susceptible strains of *Plasmodium falciparum*. Usual prophylaxis dosing is once weekly starting the week prior to departure to an endemic area and for four weeks following return [10].

There are numerous published case reports [8, 11–19] and cohort studies [20–24] in the civilian literature of individuals who have experienced neuropsychiatric reactions to the prophylactic use of mefloquine hydrochloride (Lariam). Cohort studies have indicated that the use of mefloquine is associated with an increased risk of depression [20–23], central nervous system symptoms [21], and insomnia [24]. One study found that females reported adverse events more frequently than males [20]. Severe neuropsychiatric reactions to mefloquine have primarily been reported in case studies and have included depression [8, 13, 18, 23] and psychosis [8, 12, 15–19, 25]. There have also been reports of neuropsychological side effects associated with the prophylactic use of mefloquine, including deficits in orientation, attention, psychomotor speed, and executive, visuospatial, and verbal memory functioning [26].

There have been two randomized double-blind studies of mefloquine [27, 28]. One study included 119 participants and compared mefloquine to atovaquone (250 mg) plus chloroguanide (100 mg) and found that prophylactic use of mefloquine was associated with higher scores on scales measuring depression [27]. In the only published study of US military personnel, 359 Marines were randomized to two prophylactic mefloquine regimens compared with chloroquine [28]. Depressive feelings were noted in two to three times more individuals in the mefloquine groups early in the course of the study and then resolved in most participants as tolerance developed.

The Cochrane Database review includes 516 adverse events published in 136 papers between 1976 and 2000 [29]. However, these adverse events are primarily established through spontaneously reported symptoms rather than through any large-scale surveillance system. Therefore, the authors believe that the reported 1 in 10,000 incidence of neuropsychiatric adverse effects represents a significant underreporting.

The increasing body of literature on the potential for severe neuropsychiatric reactions with the prophylactic use of mefloquine led the Food and Drug Administration (FDA) to release a Safety Alert in September 2002 [30]. This alert states “Larium should not be prescribed for prophylaxis in patients with active depression, a recent history of depression, generalized anxiety disorder, psychosis, or schizophrenia or other major psychiatric disorders, or with a history of convulsions.” Although previous case reports have been published on the neuropsychiatric reactions to mefloquine in civilians, the present case report is the first published report of a significant neuropsychiatric adverse reaction to prophylactic mefloquine in a deployed US military member.

## 2. Case Presentation

The patient was a 27-year-old male Staff Sergeant with 9 years of active-duty military service in the U.S. Air Force. He was an electronic warfare technician with an excellent service record who successfully supervised 20 military personnel prior to his deployment to an undisclosed location in Southwest Asia. In the six months prior to deployment, he received treatment by both military and civilian physicians for chronic low back pain associated with degenerative disc disease and secondary depression. He was prescribed a variety of medications including acetaminophen and codeine, hydrocodone and acetaminophen, acetaminophen and oxycodone, sertraline, mirtazapine, clonazepam, lorazepam, diazepam, and buspirone. He also participated in outpatient counseling with an Air Force social worker. 

Prior to his military deployment, he discontinued all medications except sertraline (200 mg per day) and diazepam (2 mg, as needed). When the patient was medically screened for deployment, he reported he was feeling well enough both physically and emotionally to be deployed. He was subsequently medically cleared for deployment, and shortly thereafter he received his first weekly dose of mefloquine for antimalarial prophylaxis. After arriving in the deployed location, approximately one week later, he took his second dose of mefloquine. Shortly after his second dose, he reported restlessness and sleep disturbances. The following week, after taking his third dose, the patient reported feeling moderately depressed. After taking his fourth weekly dose, he reported becoming very depressed and emotionally labile. His wife verified that he called home about this time and sounded very distraught and could not stop crying. Shortly after his fifth weekly dose, the patient began experiencing florid visual hallucinations, difficulty speaking, vivid nightmares, hypnopompic sleep paralysis, intense feelings of depression with uncontrollable crying, and strong suicidal ideations. He sought medical attention at a deployed medical clinic and was told to discontinue taking the mefloquine. He continued to take sertraline (200 mg per day), and his diazepam was increased from 2 mg as needed to 2–4 mg twice daily to manage his acute emotional distress. He was later found wandering aimlessly on the other side of the deployed base, confused, and disoriented. Over the course of his deployment, the military service member was not exposed to any activity or stressor that might cause traumatic reactions and justify the symptoms recorded.

The patient was subsequently aeromedically evacuated from his deployed location and arrived home a few days later, where his physical presentation and behavior alarmed his spouse. He was initially very quiet, nervous, and tense, displayed significant problems with word finding and speech enunciation, was afraid to go to sleep or be left alone, and was emotionally labile. The patient reported feeling overwhelmed by sensory stimuli and feeling “like a whole rush of stuff going into your brain at one time.” He perspired profusely and complained of being hot even on cold days. He continued to experience occasional visual hallucinations, violent nightmares, and “flashbacks.” He reported that on one occasion, he became enraged and held a chair over his head, as if he was going to throw it at his wife when she did not respond quickly enough after he called her. All of these behaviors were reported to be very atypical for the patient. 

He was subsequently admitted to the inpatient psychiatric unit at Wilford Hall Medical Center in San Antonio, Texas, for further evaluation. His behavior on the inpatient psychiatric ward was very labile, ranging from agitated and confrontational, to tearful and shaky, to calm and cooperative. The patient participated in a neuropsychological evaluation over the course of two days, including an extended clinical interview and the administration of a comprehensive eight-hour battery of cognitive and personality tests. He was cooperative and appeared to put forth his best effort throughout the evaluation. His performance on “effort” tests and his scores on validity scales from the Minnesota Multiphasic Personality Inventory-2 were not consistent with symptom exaggeration, inadequate effort, or malingering. 

The patient's neuropsychological evaluation was considered valid. Although his full-scale intelligence quotient (IQ) was in the average range, he displayed significant deficits on certain neuropsychological tests, including moderate to severe impairments on measures of verbal learning, auditory and visual memory, verbal productivity, and upper extremity motor speed and dexterity. Milder impairments were noted on measures of sustained attention and concentration, information processing speed, visuomotor coordination and construction, and grip strength. Personality testing indicated that the patient was moderately depressed, anxious, tense, confused, and socially alienated. He reported a higher than average number of somatic complaints, reflecting his perception that his physical health was failing and his mind was not functioning properly.

## 3. Discussion

The etiology of severe neuropsychiatric reactions to prophylactic mefloquine is not clear. It has been suggested that it may be related to primary liver damage with secondary thyroid damage [31]. Severe mefloquine-induced neuropsychiatric side effects have also been attributed to a central anticholinergic syndrome [32]. It has been suggested that adverse reactions to mefloquine, including neuropsychiatric reactions, can be potentiated by concomitant ethanol ingestion [33] and by conditions that can lead to mild dehydration such as prolonged air travel, hot or dry conditions, and strenuous physical activity [31]. In the present case study, the patient's history of depression and the concomitant use of sertraline, diazepam, and mefloquine should be considered factors which may have predisposed him for the severe neuropsychiatric reaction.

Prophylaxis against malaria still has benefits that frequently outweigh the risks, especially for deployed military medical personnel. Since 2001, approximately 2 million US military personnel have deployed to Iraq and Afghanistan, and prophylactic mefloquine continues to be used in some locations [34]. Military medical providers should be aware of the potential for neuropsychiatric reaction with the prophylactic use of mefloquine, especially in patients with a recent history of neuropsychiatric illness (mood disorders, psychotic disorders, or seizure disorders) [35]. In these cases, an alternative antimalarial agent (e.g., doxycycline) might be preferable. Additionally, patients receiving mefloquine should be educated to seek medical attention if they experience anxiety, depression, restlessness, confusion, or irritability. Additional prospective surveillance studies of evaluating the prophylactic use of mefloquine in deployed military personnel are needed.
